# Machine learning prediction of early reoperation following lower extremity tumor resection and endoprosthetic reconstruction: A PARITY trial secondary analysis

**DOI:** 10.1186/s13018-025-06139-7

**Published:** 2025-08-04

**Authors:** Nicole J. Newman-Hung, Akash A. Shah, Joseph K. Kendal, Nicholas M. Bernthal, Lauren E. Wessel, on behalf of the PARITY Investigators

**Affiliations:** 1 Department of Orthopaedic Surgery, University of California, Los Angeles, CA USA; 2 Division of Orthopaedic Surgery, Department of Surgery, University of Calgary, Calgary, Canada; 3 15th Street, Suite 3140, Santa Monica, CA 90404 USA

**Keywords:** Tumor resection, Endoprosthetic reconstruction, Reoperation, Machine learning, Prediction

## Abstract

**Background:**

Oncologic resection and endoprosthetic reconstruction of malignant bone tumors carries a high risk of complication and secondary surgery. Given the significant morbidity associated with reoperation in systemically compromised patients, accurate risk stratification is critical to patient counseling and shared decision-making. The purpose of this study was to develop a machine learning (ML) model for prediction of reoperation within one year of lower extremity tumor resection and endoprosthetic reconstruction.

**Methods:**

Using data from the PARITY trial, 54 features across 604 lower extremity endoprosthetic reconstructions were evaluated as predictors of all-cause reoperation within one year. Logistic regression (LR), Random Forest, gradient boosting, AdaBoost, and XGBoost were used for model building. Standard metrics of area under receiver operating characteristic curve (AUROC), area under the precision-recall curve (AUPRC), and Brier scores were used to evaluate model performance. Important features for the top-performing model were determined.

**Results:**

Of 604 lower extremity endoprosthetic reconstructions performed in the study period, 155 patients (25.7%) underwent at least one reoperation. The Gradient Boosting model had the highest discrimination (AUROC = 0.817, AUPRC = 0.690) of the tested models and was well-calibrated. Surgical site infection (SSI), operative time, white race, negative pressure wound therapy (NPWT) use, and female sex were the five most important features for model performance.

**Conclusions:**

We report a well-calibrated ML-driven algorithm with high discriminatory power for the prediction of all-cause early reoperation following lower extremity tumor resection and endoprosthetic reconstruction. Preventing SSI remains paramount to avoiding the morbidity of reoperation after complex oncologic limb salvage surgeries.

## Background

Malignant lower extremity bone tumors are often treated with wide surgical resection and limb-salvage with endoprosthetic reconstruction. These are complex procedures performed in medically compromised patients who often require extensive soft tissue resection and long operative times. Oncologic resection and endoprosthetic reconstructions are consequently susceptible to a variety of failure mechanisms, resulting in high reoperation rates and significant patient morbidity [1, 2].

Reducing reoperation rates begins with identifying potential risk factors that drive reoperation following endoprosthetic reconstruction after tumor resection. Prior studies have reported patient-, tumor-, and treatment-related factors that may contribute to reoperation including diabetes, tobacco use, tumor location, implant type, soft tissue involvement, and perioperative systemic therapy [3–5]. Yet these findings are significantly limited by study heterogeneity of primarily retrospective single-center data with limited clinical follow-up. As such, modifiable and non-modifiable risk factors for reoperation in this patient population remain poorly understood.

The Prophylactic Antibiotic Regimens in Tumor Surgery (PARITY) was a multicenter, prospective randomized control trial that investigated the impact of antibiotic regimen on lower extremity tumor resection and endoprosthetic reconstruction outcomes. In a recently published secondary analysis of the PARITY trial data, Kendal et al. utilized traditional multivariable statistical analysis to identify tumor type, operative time, and use of negative-pressure wound therapy (NPWT) as independent risk factors for all-cause reoperation within one year of index surgery [6]. While this hypothesis-generating analysis provided foundational knowledge of factors driving reoperation risk, leveraging novel methodologic approaches could further strengthen our understanding by providing new insights.

Machine learning (ML) represents a powerful predictive modeling tool that has been increasingly employed in medical research due to its ability to identify complex nonlinear relationships and factor-factor interactions [7]. Within orthopaedics, advanced ML methods have been applied to various spine, arthroplasty, and shoulder pathologies to develop decision-aid tools, often outperforming traditional logistic regression (LR) [8–10]. To our knowledge, ML applications in predicting early reoperation after lower extremity oncologic resection and endoprosthetic reconstruction remain limited, largely due to the lack of an adequate data source given disease rarity [11]. The primary aim of this study was to build a ML model for prediction of early all-cause reoperation following lower extremity tumor resection and endoprosthetic reconstruction. Secondarily, we sought to identify novel patient features driving prediction of early reoperation after lower extremity tumor resection and endoprosthetic reconstruction. We hypothesized that the best-performing ML model would identify novel risk factors that were not identified previously through traditional analysis. We aim to enrich preoperative patient counseling and shared decision-making by providing more accurate prognostic tools, which may be of high utility in this high-risk patient population.

## Methods

### Study design

A formal proposal for this secondary analysis was submitted to and approved by the PARITY trial investigators. Of note, our work is distinct from Kendal et al.’s previously published secondary analysis where univariate statistical analysis and multivariate Cox proportional hazards regression models with independent variables were employed. This study was conducted in accordance with guidelines from the Transparent Reporting of a Multivariable Prediction Model for Individual Prognosis or Diagnosis (TRIPOD + AI) [12].

### Data source

Following secondary analysis proposal acceptance, we obtained data from the PARITY trial. From January 2013 to October 2019, PARITY investigators enrolled 604 patients undergoing lower extremity tumor resection and endoprosthetic reconstruction across 48 sites in 12 countries [13]. Patients were randomized to receive 1 versus 5-day postoperative antibiotic regimens. For the PARTIY trial, the primary outcome measure was development of surgical site infection (SSI) within one year of surgery. Secondary outcome measures included antibiotic-related complications, unplanned additional operations, oncologic and functional outcomes, and mortality [14]. Patient demographics, medical comorbidities, tumor characteristics, diagnostic information, surgical details, and clinical outcome measures described above were collected prospectively.

### Explanatory variables and outcome measures

Our primary outcome of interest was all-cause reoperation within one year of the index surgery. Types of reoperations included irrigation and debridement, implant exchange, implant revision, amputation, flap reconstruction, repeat tumor excision, antibiotic spacer insertion, extensor mechanism reconstruction, skin grafting, fasciotomy, patellar resurfacing, and abductor mechanism reconstruction (Table 1). Fifty-four patient, tumor, and treatment characteristics were included as explanatory variables from the PARITY trial data (Table 2), including fifty-two binary/categorical variables and two continuous variables.

**Table 1:** Reoperations performed within one year of index surgery

Type of Reoperation	Reoperation Events, *n* (%)
Irrigation and debridement	96 (31.4)
Implant exchange	38 (12.4)
Implant revision	35 (11.4)
Amputation	20 (6.5)
Wound flap	13 (4.3)
Repeat tumor excision	13 (4.3)
Antibiotic spacer insertion	12 (3.9)
Extensor mechanism reconstruction	8 (2.6)
Skin grafting	7 (2.3)
Fasciotomy	2 (0.7)
Patellar resurfacing	1 (0.3)
Abductor mechanism reconstruction	1 (0.3)
Other	60 (19.6)

**Table 2:** Explanatory features included in model development

Sex
Age
Race/ethnicity
Tumor type Primary malignant bone tumor Soft tissue sarcoma Metastatic bone disease Benign aggressive bone tumor
Tumor type osteosarcoma
Tumor type giant cell tumor of bone
Tumor type non-osteogenic sarcoma of bone
Tumor type chondrosarcoma
Tumor type Ewing’s sarcoma
Tumor type soft tissue sarcoma
Tumor type other
Presence of associated soft tissue mass
Tumor location proximal femur
Tumor location middle femur
Tumor location distal femur
Tumor location proximal tibia
Tumor location middle tibia
Tumor location distal tibia
Tumor location other
Tumor location other specified
Presence of other malignancy
Presence of metastases
Diabetes mellitus
Immunocompromised state
Active smoking
Active alcohol use
History of intravenous drug use
Non-steroidal anti-inflammatory drug use
Opioid use
Anti-hypertensive medication use
Cardiac medication use
Pulmonary medication use
Osteoporosis medication use
Preoperative antibiotic use
Biopsy type
Neoadjuvant chemotherapy administration
Neoadjuvant radiation
Other neoadjuvant treatment
Neutropenia at time of surgery
Total length of incision
Presence of laminar flow in operating room
Space suit wear in operating room
Operative time
Fixation type
Prosthesis type
Use of bone graft
Area of muscle excised
Vascular reconstruction required
Intraoperative topical antibiotic use
Intraoperative intravenous tranexamic acid use
Primary closure
Local muscle/skin graft required
Local fasciocutaneous graft required
Free flap requirement
Use of negative pressure wound therapy
Use of postoperative suction drain
Use of postoperative urinary catheter
Margin status
Presence of extra-articular resection
Shared hospital room
Length of hospital stay
Surgical site infection

### Model development

We employed five standard ML benchmark models that capture different classes of ML modeling including: LR (linear classifier), random forest [15] (a tree-based ensemble classifier), AdaBoost [16], as well as Gradient Boosting [17], and XGBoost [18] (boosting ensemble classifiers). We implemented LR, Random Forest, AdaBoost, and Gradient Boosting machines using the *scikit-learn* Python library [19] while we built XGBoost using the *xgboost* Python library [18]. Model hyperparameters were chosen through grid search. For LR, the coefficient for L2 regularization was chosen from a set of values on a logarithmic scale between 1.0 x e^− 3^ and 1.0 x e^3^. For Random Forest, AdaBoost, Gradient Boosting, and XGBoost, the number of trees were selected from {50, 100, 200, 300} while the maximum depth of each tree was selected from {2, 3, 4, 5}.

### Model evaluation

After model development, we evaluated discrimination and calibration performances of each ML model. We employed five-fold stratified cross-validation to avoid overfitting. In each cross-validation fold, 80% of the study population was used to train our five ML benchmark models while the remaining 20% was held out as a testing cohort for performance evaluation.

Discrimination represents each model’s ability to distinguish patients who required early reoperation from those who did not. We assessed discrimination with area under the receiver operating characteristic curve (AUROC), which represents the probability that a model assigns a higher risk to a patient who experienced an outcome compared to a patient who did not experience the outcome. An AUROC of 0.5 indicates random prediction (no discriminative power) while an AUROC of 1 indicates perfect discrimination. An AUROC of 0.5–0.7 indicates low accuracy, 0.7–0.9 indicate moderate accuracy, and a value greater than 0.9 indicates high accuracy [20].

Calibration reflects how well the model’s predictions align with the actual outcomes within the study population. We assessed calibration with the calibration slope and calibration intercept. The calibration slope is a measure of prediction spread by the model; a slope of 1 is consistent with perfect spread. A calibration intercept close to 0 indicates minimal overestimation or underestimation of an outcome by the model [21, 22]. We additionally assessed discrimination and calibration with the Brier score, which is equivalent to the mean squared error. Brier scores closer to zero indicate lower deviation of a model’s predictions from observed outcome probability.

We also determined the area under the precision-recall curve (AUPRC), which is useful in unbalanced datasets when negative cases far outnumber positive cases such as our cohort. The precision-recall (PR) curve reflects the tradeoff between positive predictive value (precision) and sensitivity (recall). Unlike AUROC, which assesses the model’s ability to discriminate between positive and negative cases, the PR curve represents the model’s ability to correctly identify positive cases while ignoring true negatives, which comprise most cases in the cohort [23, 24]. In contrast to AUROC, where the baseline value represents random prediction, the AUPRC baseline value represents the proportion of true positives in the cohort. An ideal classifier has an AUPRC of 1 and correctly identifies all positive cases, achieving perfect recall, while avoiding any misclassification of negative cases, achieving perfect precision. For AUPRC, random prediction results in the baseline value. Greater deviation of AUPRC from the baseline value reflects a model that can better handle positive cases.

### Feature importance

We employed a partial dependence function to evaluate the significance of a specific feature in influencing model performance [17]. Through this approach, we analyzed the average impact on predicted risks when a given feature’s value is changed.

## Results

### Cohort characteristics

Of 604 lower extremity endoprosthetic reconstructions performed in the PARITY trial, 155 patients (25.7%) underwent cumulative 306 reoperation events within one year of the index operation. Irrigation and debridement was the most frequently performed reoperation (31.4%). The full distribution of reoperation types is demonstrated in Table 1. Overall cohort characteristics including patient demographics and highlighted medical comorbidities are summarized in Table 3.

**Table 3:** Cohort characteristics with selected medical comorbidities and treatment details

Characteristic	Total (*n* = 604)
Age, mean (SD), y	41.2 (21.9)
Sex	
Male Female	361 (21.9) 243 (40.2)
Race and ethnicity	
Asian Black Hispanic Indigenous White Other Unknown	114 (18.9) 43 (7.1) 34 (5.6) 15 (2.5) 384 (63.8) 12 (2.0) 2
Systemic metastases	
No Yes	499 (82.6) 105 (17.4)
Adjuvant therapy	
No Yes	295 (48.8) 309 (51.2)
Location of tumor	
Tibia Femur	108 (17.9) 496 (82.1)
Type of tumor	
Bone tumor Soft tissue sarcoma Oligometastatic disease	486 (80.5) 62 (10.3) 56 (9.3)
Neutropenia at time of surgery	
No Yes	465 (82.9) 96 (17.1)
Operative time, median (Q1-3), min	270 (205–377)
Surgical site infection	96 (15.9%)
Length of stay, median (Q1-3), days	6 (5–8)

### Model performance and calibration

**Fig. 1:**
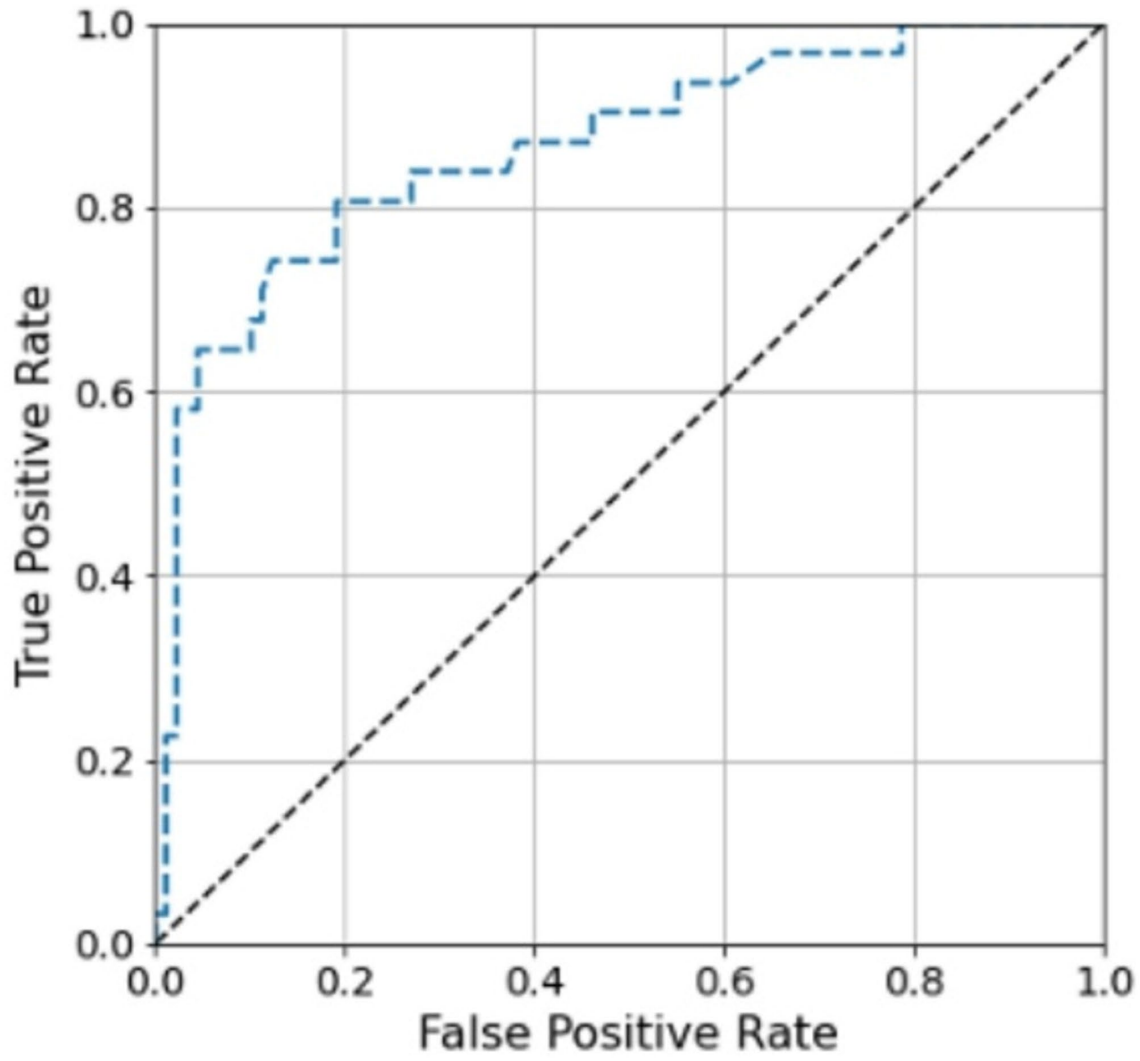
Receiver Operating Characteristic (ROC) Curve for highest-performing model (Gradient Boosting)

Gradient Boosting demonstrated the highest discrimination of all tested models (AUROC 0.817 *±* 0.04). The Gradient Boosting model was also well-calibrated with a calibration slope of 1.15 and calibration intercept of -0.04. The Brier score for the Gradient Boosting model was 0.130 *±* 0.020, compared to a null model Brier score of 0.193. Additionally, Gradient Boosting had the highest AUPRC of 0.690, which is compared against a random classifier of 0.257 for this cohort (positive case proportion). The AUROC, AUPRC, and Brier scores for all tested models are shown in Table 4. Traditional LR outperformed AdaBoost (AUROC 0.783 versus 0.718, AUPRC 0.642 versus 0.523, Brier Score 0.138 versus 0.183) but otherwise performed worse than all other tested ML models. The receiver operating characteristic curves of the Gradient Boosting model is shown in Fig. 1.

**Table 4:** Discrimination and calibration

Model	AUROC	AUPRC	Brier Score
Logistic Regression	0.783 $\:\pm\:\:$0.04	0.642 $\:\pm\:$ 0.10	0.138 $\:\pm\:$ 0.019
XGBoost	0.806 $\:\pm\:\:$0.03	0.674 $\:\pm\:\:$0.08	0.135 $\:\pm\:\:$0.016
Gradient Boosting	0.817 $\:\pm\:\:$0.04	0.690 $\:\pm\:\:$0.09	0.130 $\:\pm\:\:$0.020
AdaBoost	0.718 $\:\pm\:\:$0.04	0.523 $\:\pm\:\:$0.08	0.183 $\:\pm\:\:$0.02
Random Forest	0.815 $\:\pm\:\:$0.04	0.651 $\:\pm\:\:$0.08	0.137 $\:\pm\:\:$0.02

### Relative feature importance

Development of SSI, white race, female sex, NPWT use, and opioid use were the five most important binary features while operative time, length of stay, and patient age were the most important continuous predictive feature in Gradient Boosting. The relative importance of the top ten explanatory features to the performance of Gradient Boosting are shown in Table 5.

**Table 5:** Relative feature importance for gradient boosting

Binary Features	Rank in Gradient Boosting	Change to Risk Prediction
Surgical site infection	1	0.5755
White race	2	0.0535
Female sex	3	0.0479
NPWT use	4	0.0427
Opioid use	5	0.0286
Giant cell tumor of bone	6	0.0220
Osteosarcoma	7	0.0146
Open biopsy	8	0.0044
**Continuous Features**		
Operative time	1	0.1073
Length of stay	2	0.0499
Age	3	0.0463

## Discussion

With high rates of postoperative infection (15.9%) and all-cause early reoperation (25.7%), patients undergoing lower extremity oncologic resection and endoprosthetic reconstruction remain a high-risk patient population [14, 25]. As such, reducing the morbidity and costs associated with reoperation is of paramount importance. Until recently, our understanding of risk factors for reoperation was based on small, heterogeneous, and retrospective cohorts [2]. In a secondary analysis of the PARITY trial using traditional statistical methods, Kendal et al. found that primary malignant bone tumor type, operative time, and use of NPWT at time of index surgery were associated with early reoperation [6].

Utilizing ML may enrich our understanding of modifiable and non-modifiable risk factors previously identified through traditional analysis by detecting complex, non-linear relationships and interactions between explanatory features [26, 27]. The purpose of this study was to develop a ML model to predict all-cause reoperation within one year of undergoing lower extremity tumor resection and endoprosthetic reconstruction. We have developed a Gradient Boosting model that is well-calibrated and predicts reoperation within one year with excellent discrimination. Compared to previously performed traditional regression analyses, we identified additional novel demographic and clinical variables that contributed to reoperation prediction [6].

While multivariable LR has long been the preferred method for outcome prediction, ML has emerged as a promising predictive modeling tool for clinical outcomes in orthopaedic surgery, often outperforming traditional LR [28, 29]. These studies span multiple subspecialties, frequently utilizing large national or regional databases [8, 9, 30–37]. Yet ML applications to orthopaedic oncology remain relatively limited, largely due to a paucity of high-quality data sources. Deep learning models may increase data points for model development by integrating radiomics (imaging) and pathomics (pathology). Recently, deep learning models have been created for soft tissue sarcoma diagnosis and management [38, 39]. Within the osteosarcoma literature, deep learning models have been developed to assess degree of tumor necrosis after chemotherapy from digitized pathology, to predict survival from RNA sequencing classification, and to predict metastatic disease development from clinical features [38, 40, 41]. While these ensemble models offer incredible potential in personalized risk prediction, ML models that exclusively use clinical data may be more accessible and easier to externally validate.

To our knowledge, there has only been a single published comparative ML-driven model to predict early reoperation following oncologic resection and endoprosthetic reconstruction [11]. Yet, the present study remains novel as we employed unique algorithmic development approaches with unique benchmark ML models, different input variables, and distinct performance metrics compared to Deng et al. [11]. Given the rapid rise of ML-based methodology in recent years, there is a growing emphasis on investigating rigor and reproducibility within the field [42, 43]. Nuances in data sources, feature selection, model hyperparameter tuning, and performance metrics can lead to novel conclusions, highlighting the need for careful interpretation of ML literature as the methodology becomes more commonplace. As such, we believe our study meaningfully contributes to the growing body of predictive ML modeling within orthopaedic oncology. In the present study, we developed a Gradient Boosting model that predicts early reoperation with excellent discrimination. Additionally, we identified the factors most important for model performance. The most important binary feature for Gradient Boosting performance was SSI development.

SSI was the most important binary feature for model performance. As defined by the PARITY registry, SSI included superficial incisional, deep incisional, and organ/space infections. SSI remains a frequently encountered postoperative complication as infection rates after lower extremity endoprosthetic reconstruction are increased compared to after non-oncologic conventional arthroplasty (13% compared to 0.5-2%) [13, 44, 45]. Several surgical and medical strategies have been proposed for SSI prophylaxis. Possible surgical interventions to prevent SSI include topical antibiotic use, silver and antibiotic-coated implants, surgical space suit wear, use of laminar flow in the operating room, and NPWT use; however, these modalities have shown mixed effects on SSI reduction [46–49]. There remains debate on the association between medical therapy and SSI development. For example, while some studies have shown increased rates of SSI with neoadjuvant chemotherapy administration, others have demonstrated that neoadjuvant chemotherapy does not impact wound infection rates or deep infection rates [50–52]. Consistent with Kendal et al.’s analysis, neoadjuvant chemotherapy administration was not a significant predictor of early reoperation in this study. In terms of perioperative care, postoperative drain use for ≥14 days was an independent predictor of SSI development after lower extremity tumor surgery (HR 3.6) [53]. Ultimately, a multidisciplinary approach to preoperative medical optimization, systemic treatment administration, and postoperative care offers a comprehensive strategy for preventing SSI, which is crucial for avoiding early reoperation.

Notably, we also found that NPWT use at time of index surgery and operative time were among the most important predictive features. These findings are in line with Kendal et al.’s findings in their multivariable regression analysis and with Deng et al.’s ML analysis [6, 11]. In this patient population, increased reoperation risk with NPWT use may reflect a tenuous soft tissue envelope. Skin grafting versus free flap coverage may be considered in preoperative planning for these patients. Intraoperative efficiency is particularly important in this often-immunocompromised patient population, as Gazendam et al. demonstrated that increased operative time the only significant predictor of SSI and reoperation in patients who received neoadjuvant chemotherapy prior to undergoing lower extremity endoprosthetic reconstruction [50]. This finding also reflects prior reports from elective arthroplasty literature where increased operative time was associated with reoperation (OR 1.05 with every 10-minute increase) [54]. Contrary to Kendal et al. who found that benign aggressive tumor type was associated with lower risk of early reoperation (HR 0.15) and Deng et al. who identified metastatic bone disease as a predictor of reoperation, we identified giant cell tumor of bone as the sixth most important feature contributing to model performance; osteosarcoma was the seventh most important binary feature [6, 11]. Non-modifiable, demographic-based variables including female sex and white race were also identified as contributory features to model performance. Finally, we identified opioid use as a modifiable predictive feature, which underscores findings in elective total knee arthroplasty where preoperative opiate use was associated with increased risk of early revision (OR 1.40) [55].

### Limitations

The greatest strength of our study lies in the high-quality data used for model development, as the PARITY trial cohort included prospectively collected granular data across nearly 50 sarcoma centers internationally. Yet our study has multiple limitations. First, there is a risk for overfitting present with any predictive modeling approach. Although we attempt to protect against overfitting with our derivation and validation strategies, external validation studies are necessary to determine the generalizability of the reported model. Additionally, while the reported model can improve prediction accuracy, it is not explanatory. ML models are optimized for prediction, not explanation. For example, SSI had the greatest impact on model performance; however, as a postoperative event, SSI is not known at the time of surgical planning and thus has limitations in directly informing preoperative risk stratification. This reflects an inherent limitation to ML modeling, which prioritizes predictive accuracy over temporal or causal explanations. Additionally, we were unable to predict specific reoperation types primarily due to the low event rate across categories. There are certainly different clinical implications for infectious, mechanical, and oncologic-related reoperations. Future studies with larger cohorts and sufficient events in reoperation categories are needed to better inform targeted prevention efforts. Furthermore, while predicting early reoperation is of great clinical utility, predicting long-term reoperation would also offer valuable insight into implant longevity and specific failure mechanisms. Given the PARITY data source is limited to one year follow-up, we were unable to assess more long-term endpoints. Finally, external validation studies are necessary to determine the generalizability of the reported model.

## Conclusions

In this secondary analysis of data from the PARITY trial, we demonstrate that utilizing ML modeling enables the accurate prediction of reoperation within one year of lower extremity tumor resection with endoprosthetic reconstruction. Our best-performing model is well-calibrated with high discriminatory power and outperforms traditional LR. Our work also emphasizes how preventing SSI after lower extremity tumor resection and endoprosthetic reconstruction is critical for avoiding early reoperation. Our findings show that while ML modeling offers a promising foundation for accurate prognostic tool development, careful interpretation is important for maximizing the benefit of these novel modeling approaches.

## Data Availability

Data and materials were provided by the PARITY trial committee after approval of our secondary analysis proposal. This data is not publicly available due to institutional policies and privacy considerations for patient confidentiality. Data may be requested through a formal secondary analysis proposal to the PARITY trial adjudication committee.
